# Remote coral reefs can sustain high growth potential and may match future sea-level trends

**DOI:** 10.1038/srep18289

**Published:** 2015-12-16

**Authors:** Chris T. Perry, Gary N. Murphy, Nicholas A. J. Graham, Shaun K. Wilson, Fraser A. Januchowski-Hartley, Holly K. East

**Affiliations:** 1 Geography, College of Life and Environmental Sciences, University of Exeter, Exeter, UK; 2 Lancaster Environment Centre, Lancaster University, Lancaster LA1 4YQ, UK; 3 ARC Centre of Excellence for Coral Reef Studies, James Cook University, Townsville, QLD 4811, Australia; 4 Department of Parks and Wildlife, Kensington, Perth, Western Australia 6151, Australia; 5 School of Plant Biology, Oceans Institute, University of Western Australia, Crawley, Western Australia 6009, Australia

## Abstract

Climate-induced disturbances are contributing to rapid, global-scale changes in coral reef ecology. As a consequence, reef carbonate budgets are declining, threatening reef growth potential and thus capacity to track rising sea-levels. Whether disturbed reefs can recover their growth potential and how rapidly, are thus critical research questions. Here we address these questions by measuring the carbonate budgets of 28 reefs across the Chagos Archipelago (Indian Ocean) which, while geographically remote and largely isolated from compounding human impacts, experienced severe (>90%) coral mortality during the 1998 warming event. Coral communities on most reefs recovered rapidly and we show that carbonate budgets in 2015 average +3.7 G (G = kg CaCO_3_ m^−2^ yr^−1^). Most significantly the production rates on *Acropora*-dominated reefs, the corals most severely impacted in 1998, averaged +8.4 G by 2015, comparable with estimates under pre-human (Holocene) disturbance conditions. These positive budgets are reflected in high reef growth rates (4.2 mm yr^−1^) on *Acropora*-dominated reefs, demonstrating that carbonate budgets on these remote reefs have recovered rapidly from major climate-driven disturbances. Critically, these reefs retain the capacity to grow at rates exceeding measured regional mid-late Holocene and 20th century sea-level rise, and close to IPCC sea-level rise projections through to 2100.

Warming sea waters, ocean acidification and rising sea levels are effects of climate that pose a major threat to coral reefs globally^1^,^2^. At a local scale, reefs are also under ever-growing pressure from multiple direct human disturbances, including eutrophication and over-fishing, as well as disease outbreaks^3^,^4^. The net effect of these cumulative disturbances has been to fundamentally change reef ecology, with many reefs exhibiting reduced coral cover, altered coral and reef-associated species abundances, and diminished structural complexity^5^,^6^,^7^. On many disturbed reefs these changes are also now impacting the carbonate budgets of reefs^8^, defined as the balance between the rate at which carbonate is produced by corals, coralline algal and other carbonate producing processes, set against the rate at which carbonate is either denuded by biological erosion (‘bioerosion’), removed by physical processes, or chemically dissolved^9^. Where carbonate budgets are positive, reefs can maintain their physical three-dimensional structures and sustain high growth potential. However, under conditions of diminished production or increased bioerosion, carbonate budgets can become net negative, limiting reef growth and leading to reef structural collapse^10^. Such fundamental changes have now occurred across much of the Caribbean, where coral cover loss and community composition changes have caused both carbonate production and erosion rates to decline^11^,^12^, significantly diminishing reef growth potential^8^. Similar trajectories are predicted for many reefs globally due to local disturbances and exacerbated by climate change^2^,^13^,^14^.

Whilst many reefs have succumbed to the combined impacts of climate- and human-driven pressures, there remain regions largely free of direct human pressures due to their geographic remoteness. This raises the question of whether such reefs are better able to cope with global climate change impacts. The geographically isolated Chagos Archipelago, central Indian Ocean (Fig. 1), is one such region. It remains, with the exception of Diego Garcia, remote from direct human disturbance, and has fish populations that can be considered semi-pristine^15^. While the mass coral bleaching event of 1998 caused severe damage to reefs in Chagos, they have demonstrated an impressive capacity for ecological recovery^16^. Here we use measured rates of both gross carbonate production and bioerosion from 28 reefs across 5 atolls in Chagos to determine their net biological carbonate budgets (G, where G = kg CaCO_3_m^−2^ yr^−1^), and use these data to address two important questions: 1) have these reefs proved sufficiently resilient to recent climatic disturbances to regain rates of carbonate production and erosion that can be considered typical of undisturbed Indo-Pacific reefs? 2) do these reefs retain the capacity to accrete at rates sufficient to offset projected increases in sea level?

## Results

Net carbonate production rates across all reefs averaged +3.7 G, with rates at the individual reef scale ranging from +9.8 to −5.0 G. Most reefs (25/28, 89%) had net positive budgets, and eight (29%) had budgets exceeding 5 G. Only 3 of the 28 reefs had net negative budgets (range: −0.3 to −5.0 G). There was, however, considerable heterogeneity in net G (Fig. 2A), with significant differences among reefs (F_24,82_ = 8.584, p < 0.001), although not among atolls (F_3,82_ = 2.192, p = 0.113). To explore the influence of wave exposure on carbonate budgets we compared averaged net and gross rates of production and erosion for sheltered and exposed reefs within each atoll, grouped based on wave energy regime. Average net G ranged from +0.2 to +6.0 G (Fig. 2A), with highest averages on reefs around both the ‘sheltered’ and ‘exposed’ margins of Peros Banhos (averages of +6.0 and +5.6 G respectively), from the ‘sheltered’ margin of Salomon (+5.0 G), and wave exposed sites on the Gt. Chagos Bank and Egmont (+5.4 G). Lowest net production rates were measured around the sheltered margins of Gt. Chagos Bank (+0.2 G) (Fig. 2A). Net G did not differ between sheltered and exposed sites within most atolls, the exception being the Gt. Chagos Bank, where exposed sites had significantly higher net G (atoll x exposure F_3,21_ = 6.054, p = 0.004).

The generally high and positive carbonate budgets are reflected in high rates of gross carbonate production (Fig. 2B) and bioerosion (Fig. 2C). Gross carbonate production averaged +6.6 G across all reefs, but differed significantly among reefs (F_24,82_ = 7.165, p < 0.001). Highest production rates were measured at Ile Poule on the sheltered margin of Peros Banhos (+11.2 G), and the lowest at Nelson at the northern margin of the Gt. Chagos Bank (+1.3 G), a reef with very low live coral cover (3.6%) as a result of recent (2015) Crown-of-Thorns starfish predation. Grouped- exposure/atoll average production ranged from +2.4 to +9.5 G (Fig. 2B), and significantly higher gross production rates were measured on the exposed compared to the sheltered Gt. Chagos Bank/Egmont reefs, while on Salomon production was significantly higher at the sheltered reefs (atoll x reef F_3,21_ = 7.689 , p = 0.001). Bioerosion rates averaged 3.4 G across all reefs, with grouped-exposure/atoll averages ranging from −2.0 to −4.2 G (Fig. 2C), though bioerosion did not vary significantly with exposure (F_1,21_ = 0.213, p = 0.673). Bioerosion rates were significantly different between reefs (F_24,82_ = 249.580, p < 0.001), but not between atolls (F_3,82_ = 1.212, p = 0.326). Highest rates were measured at Petite Coquillage, Peros Banhos (7.4 G) and the lowest at East Island, Diego Garcia (1.4 G) (see Supplementary Table S1).

**Figure 1:**
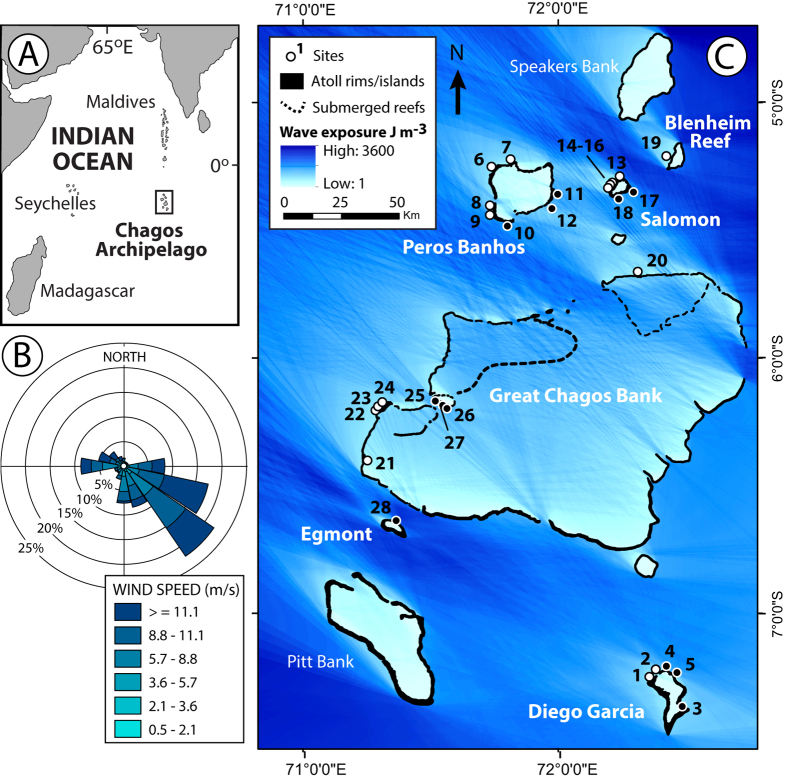
Study sites and wave energy regimes. (**A**) Location of the Chagos archipelago in the central Indian Ocean. Redrawn using Adobe Illustrator Version CS5 from Global and Planetary Change, Vol 82–83, R. P. Dunne *et al.* Contemporary sea level in the Chagos Archipelago, central Indian Ocean, p. 25–37, Copyright 2011, with permission from Elsevier; (**B**) Rose diagram showing the frequency and speed of winds (m s^−1^) affecting Chagos plotted based on the direction from which winds are generated. Plots based on hourly wind measurements from 1973 to 2001 obtained from Diego Garcia airport (n = 219,943); (**C**) Major islanded atolls (bold white font) and submerged platforms of the Chagos Archipelago and modelled wave exposures (Joules m^−3^). Numbered circles denote study sites, white fills are defined as ‘sheltered’ (<1000 J m^−3^), black fills as ‘exposed’ sites (>1000 J m^−3^): Diego Garcia - 1) Cannon Point, 2) Middle Island, 3) Horsborough Bay, 4) East Island, 5) Barton Point; Peros Banhos – 6) Ile Diamante, 7) Ile de la Passe, 8) Ile Poule, 9) Ile Gabrielle, 10) Ile Fouquet, 11) Grand Coquillage, 12) Petite Coquillage; Salomon – 13) Ile de Passe, 14) Ile Anglais north, 15) Ile Anglaise middle, 16) Ile Anglaise south, 17) Ile Takamaka, 18) Ile du Sel; Blenheim – 19) Blenheim west; Gt. Chagos Bank – 20) Nelson Island, 21) Danger Island, 22) Eagle Island south, 23) Eagle Island middle, 24) Eagle Island north, 25) Middle Brother, 26) South Brother west, 27) South Brother east; Egmont – 28) Egmont north east. Map generated in ArcMap 10.2.2 (www.esri.com/). Atoll outlines were imported into ArcMap from the Millennium Coral Reef Mapping Project (UNEP-WCMC), which is publicly available data on the web at the Institute for Marine Remote Sensing (University of South Florida) website (http://imars.usf.edu/MC/index.html). The dataset comprises 3 main components: (1) Millennium Coral Reef Mapping Project validated maps provided by the Institute for Marine Remote Sensing, University of South Florida (IMaRS/USF) and Institut de Recherche pour le Développement (IRD, Centre de Nouméa), with support from NASA; (2) Millennium Coral Reef Mapping Project unvalidated maps provided by the Institute for Marine Remote Sensing, University of South Florida (IMaRS/USF), with support from NASA. Unvalidated maps were further interpreted by UNEP-WCMC. Institut de Recherche pour le Développement (IRD, Centre de Nouméa) do not endorse these products; (3) Other data have been compiled from multiple sources by UNEP-WCMC and the WorldFish Centre in collaboration with WRI and TNC.

**Figure 2:**
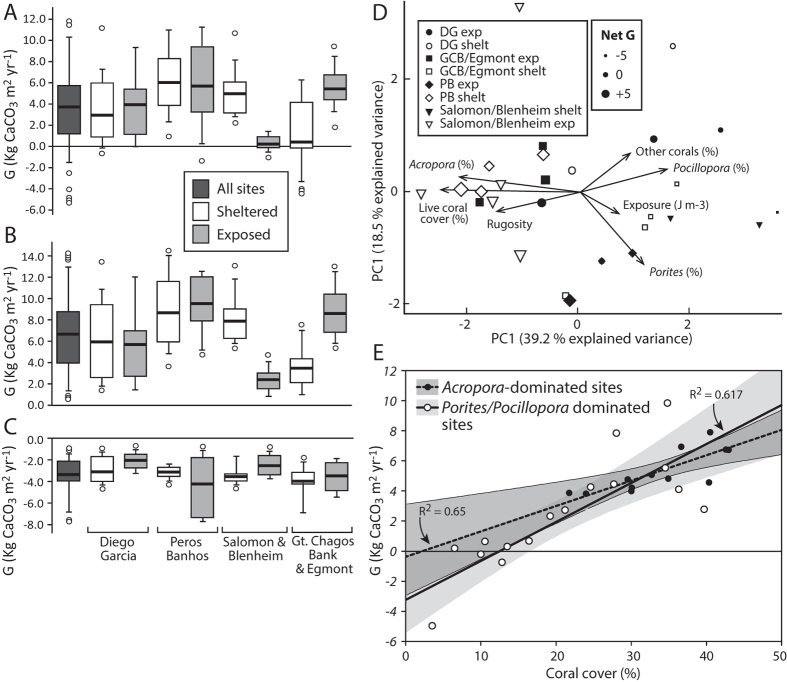
Net and gross carbonate production and erosion rates, and relationships to setting and exposure regime. Box (median and 50% quantile) and whisker (95% quantile) plots showing (**A**) net and (**B**) gross carbonate production rates, and (**C**) bioerosion rates for all sites and within each atoll. Atoll sites are grouped into “sheltered” and “exposed”; (**D**) Patterns in the net carbonate budgets of individual Chagos reefs assessed by correlation-based principle components analysis of log(*x* + 1) transformed and normalized environmental data. Eigenvectors of each ecological and physical variable are overlaid; (**E**) The linear regression and 95% confidence interval for the relationship between coral cover and the net reef carbonate budget at sites across Chagos. Sites are differentiated into those that are either *Acropora*-dominated, or *Porites*/*Pocillopora*-dominated.

To understand which taxa are driving between-site variations in carbonate budgets we assessed net G as a function of coral taxa abundance, reef structure and exposure regime. As wave exposure increased the relative abundance of more robust taxa, but especially of *Porites* sp. and *Pocillopora* (mainly *P. eydouxi*), typically increased (Fig. 2D). These taxa thus dominate on most high wave exposure reefs (Fig. 1C), which generally have lower net G rates (Fig. 2D). In contrast, high net G rates are associated with sites defined by reduced wave exposure and an increased relative abundance of tabular and branched *Acropora* spp. (Fig. 2D). Whilst sites dominated by *Porites* spp. and *Pocillopora* spp. exhibit a wide range of net budget states, we note that high positive budgets are a defining feature of reefs where carbonate production is driven primarily by *Acropora* spp. (Fig. 2E). Furthermore, we note that whilst all *Acropora*-dominated sites are likely to retain net positive budgets unless coral cover falls below only a few %, that the transition into net negative states will occur at much higher coral cover levels where *Porites* and *Pocillopora* dominate (Fig. 2E), reflecting their lower calcification and extension rates^17^.

## Discussion

**Figure 3:**
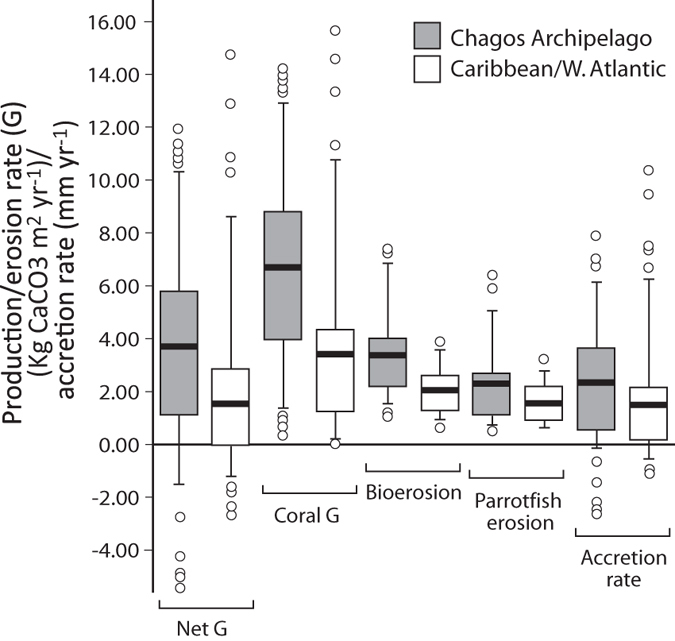
Comparisons between Chagos and the Caribbean. Box (median and 50% quantile) and whisker (95% quantile) plots showing regional differences in net G, coral G, bioerosion G, parrotfish G, and in accretion rates (mm yr^−1^) between Chagos and the Caribbean. Caribbean data from Perry *et al.*^8^,^11^,^12^.

Our estimates of contemporary carbonate production and bioerosion across Chagos indicate that most reefs have responded positively to the climate-driven mortality of 1998 in terms of their carbonate budgets. The 1998 bleaching event resulted in the mortality of ~90% of corals down to ~15 m depth in the northern atolls, and to >40 m depth around Diego Garcia^18^, a pattern repeated on many Indian Ocean reefs^19^,^20^. Shallow and mid-depth branching species, particularly *Acropora palifera* and table corals including *Acropora cytherea*, were especially impacted^21^,^22^. Since 1998, however, coral cover has recovered relatively rapidly at most sites and coral cover was restored to 1996 levels by 2010^23^. At the time of the present study (early 2015) we found that this coral recovery was reflected in generally high positive carbonate budgets on most reefs, with a third of surveyed reefs having net budgets in excess of 5 G. In addition, we note that our measured high coral production rates, which average around 6.9 G across sites, and especially those reported from the *Acropora*-dominated reefs (average 8.4 G), are close to the production rates (range ~5 to 9 G) reported as typical for undisturbed *Acropora*-dominated Indo-Pacific fore-reef settings^24^. Thus, whilst it is reasonable to assume that the 1998 event would have significantly diminished the budgets of most Chagos reefs, and especially those previously dominated by *Acropora* spp., contemporary rates have now recovered to be close to optimal for Indian Ocean reefs. This contrasts directly with the fundamental budget changes that persist at post-disturbance sites across the Caribbean, such that average carbonate production and erosion rates are higher (28% and 40% respectively) in Chagos relative to the Caribbean, and with particularly high coral production rates resulting in net G being twice as high compared to the Caribbean (Fig. 3).

We also note that on most reefs around Chagos carbonate production is predominantly driven by the same suite of coral genera; *Acropora*, *Porites* and *Pocillopora*, that dominated prior to the 1998 bleaching^22^. The relative abundance of these genera differs among sites, largely as a function of exposure regime, but they collectively contribute >70% on average of the coral carbonate being produced. We thus find no evidence for the persistence of widespread coral community changes such as have occurred on reefs across the Caribbean, and at other less remote Indo-Pacific sites^25^,^26^,^27^. In the Caribbean these changes have resulted in shifts towards coral species that were not historically important framework builders^12^, a change that has long-term implications for reef functionality because these “novel” community states may have a high probability of persisting into the future^28^,^29^,^30^, and thus of locking reefs into lower carbonate budget states. However, whilst such transitions have not widely occurred on Chagos, ecological models predict any increase in extensive mortality events would have the capacity to drive such transitions^31^. It is thus pertinent to note the recent reports of partial mortality of colonies of *A. cytherea* at some Chagos sites^32^, a fact noted in our own studies in 2015, and that some sites have also been impacted in recent years by COTS outbreaks^33^. Of most immediate concern however is the potential for another major bleaching-induced die-off, with the widespread bleaching reported during the mid-2015 sea-surface temperature anomaly event a clear indicator of the potential for such events to re-occur.

**Figure 4:**
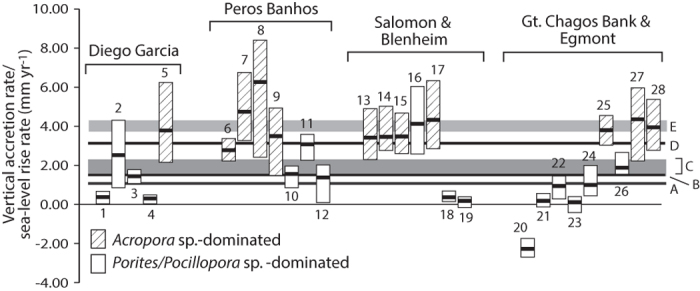
Reef accretion potential around Chagos and sea level trends and projections. Mean (black bar) and the maximum and minimum potential vertical accretion rates (mm yr^−1^) for each site in Chagos (sites are ranked by increasing wave exposure within atolls), and shown relative to global and regional sea-level rise rates as follows: (**A**) Holocene trend from ~7,500 − 3,000 yBP^45^; (**B**) Average global rise rate over last 50 years (Church *et al.* 2013)^46^; (**C**) Range of measured rates around Chagos 1993 to 2011^43^,^44^; (**D**) Average global rise rate over last 20 years^35^; E. Average predicted rise rate for the central Indian Ocean by 2100 under RCP 4.5^35^ and accounting for regional wind stress^35^. Site numbers as follows: 1. Cannon Pt, DG; 2. Middle Isld; DG, 3. Horsborough Bay, DG; 4. East Island, DG; 5. Barton Pt, DG; 6. Ile Diamante, PB; 7. Ile de la Passe, PB; 8. Ile Poule, PB; 9. Ile Gabrielle, PB; 10. Ile Fouquet, PB; 11. Grand Coquillage, PB; 12. Petite Coquillage, PB; 13. Ile Anglaise south, Sal; 14. Ile Anglaise middle, Sal; 15. Blenheim; 16. Ile Anglaise north, Sal; 17. Ile de Passe, Sal; 18. Ile du Sel, Sal; 19. Ile Takamaka, Sal; 20. Nelson, GCB; 21. Danger, GCB; 22. Eagle south, GCB; 23. Eagle middle, GCB; 24. Eagle north, GCB; 25. Egmont; 26. Middle Brother, GCB; 27. S. Brother west, GCB; 28. S. Brother east, GCB.

Arising from these observations are two important and inter-related questions about the maintenance of reef structures under future climate change. Firstly, what are the implications of the generally rapid coral community rebound around Chagos for reef growth potential? Secondly, what capacity does this instil in these reefs to respond positively to rising sea levels? Evidence from the Caribbean suggests that reef growth potential in that region has diminished, with accretion rates (mm yr^−1^) an estimated order of magnitude lower than that measured in regional Holocene core records for equivalent depth assemblages^8^. In contrast, maximum potential accretion rates around Chagos average 2.3 mm yr^−1^ across all sites, but are higher (mean: 4.2 mm yr^−1^) within *Acropora*-dominated sites, compared to *Porites*/*Pocillopora* dominated sites (mean: 0.9 mm yr^−1^) (Fig. 4). Considerable between-site/atoll variability is also evident, with highest rates occurring on reefs along the sheltered margins of Peros Banhos (4.30 mm yr^−1^) and Salomon (3.74 mm yr^−1^), and on the Brothers reefs (Gt. Chagos Bank; 3.36 mm yr^−1^) (Fig. 4). The lowest rates occur at those few sites (Nelson Island, and along the western margins of the Gt. Chagos Bank) where localised COTS outbreaks have caused recent coral mortality^33^. Viewed in the context of Indian Ocean Holocene reef growth, where shallow water accretion rates have averaged ~3.1 mm yr^−1^ over the last ~6,500 years^34^, our datasets suggest that the current prognosis in terms of reef growth potential around Chagos, and especially for the *Acropora*-dominated reefs, is positive.

Predicting reef structural integrity and growth potential under present and future sea-level rise scenarios is more problematic and depends on the interaction between reef accretion potential (driven partly by reef ecology) and sea-level rise rates. A comparison of our accretion rate estimates against recent regional sea-level trends indicates that many Chagos reefs, including all those dominated by *Acropora* spp., should have the potential to accrete at rates above those measured using sea level altimetry data over the period 1950 to 2000 (Fig. 4). Projecting into the future, the IPCC AR5 report projects a global mean sea-level rise rate by 2081-2100 of a little above 4 mm yr^−1^ for the central Indian Ocean under scenario RCP 4.5^35^, and accounting for regional wind-stress^36^. These rates are close to the average potential vertical accretion rates measured across our *Acropora* dominated sites (~4.2 mm yr^−1^), suggesting that even under these elevated rise rates that most *Acropora*-dominated reefs have the potential to closely track rising sea-levels over the coming century. However, higher rates predicted under RCP 6.0 and 8.5 scenarios would lead to a slight deepening (in the order of a few decimetres) over the fore-reef slope habitats by 2100.

There are, however, two important caveats that need consideration here. The first is that *Acropora* sp. tend to be highly susceptible to episodic disturbances^26^,^27^ and, as noted, localised partial mortality has recently impacted colonies of *A. cytherea*^32^. Thus carbonate budgets (and resultant accretion rates) may be inherently more dynamic over time on reefs dominated by such corals, compared to those where *Porites* and *Pocillopora* spp. drive carbonate production. Long-term potential accretion rates may therefore not be so different between the two, especially where coral cover and thus carbonate production (see Fig. 3E), is high. However, an additional contributing factor will be the rate of physical removal of framework which will, to varying degrees, reduce reef accretion. There is little data with which to parameterise models of physical reef framework removal but, using data on background rates from the Maldives^38^, albeit from a different reefal setting, a reasonable estimate is that along sheltered reef margins about 20% of annual framework produced may be lost (compared to >50% along exposed margins). Assuming similar attrition rates this would lower average accretion on *Acropora*-dominated reefs to ~2.9 mm yr^−1^ (range 2.3 to 4.5 mm yr^−1^). Thus, even accounting for framework removal the best available estimates would suggest that most ‘sheltered’ reefs will retain the capacity, providing *Acropora* cover is maintained, to accrete at rates above those measured across the region over the last ~50–60 years, and within or close to the rates projected for the next 100 years (Fig. 4). In contrast, it seems reasonable to assume that a high proportion of the carbonate produced from the upper shelf areas along the exposed atoll margins will be removed by physical processes. Indeed, we note that all the exposed margin sites lack Holocene framework, suggesting a situation of long-term carbonate export dominance in these settings. However, we also note that this has not previously inhibited reef flat and reef island development on these high energy margins, and thus conclude that continued coral health and carbonate productivity along these exposed margins will remain essential for supplying rubble and sediment^39^ to maintain these reef flats and islands.

Collectively, these findings highlight the capacity of the Chagos reefs, which are both geographically remote and isolated from compounding human impacts, to not only recover their ecological and geomorphic functions relatively rapidly following major past climate-driven perturbations, but also to retain the capacity to respond positively to future increases in sea level. Clear differences in accretion potential occur between sites, largely reflecting reef ecology and wave exposure regimes, but the highest accretion rates presently occur predominantly on those reefs where *Acropora*-drives carbonate production, and which are mostly along sheltered atoll margins. A key implication of this is that the capacity of reefs to track projected sea-level rise is generally lost (and certainly threatened) in the absence of *Acropora*, although we must assume, given the higher susceptibility of *Acropora* spp. to episodic disturbance, that the accretion potential of such reefs may inherently fluctuate over time. This raises an important note of warning in relation to any increase in the magnitude and frequency of disturbance events, since these would most likely preferentially impact those coral taxa with the highest growth rates^17^, and which thus have the greatest capacity to maintain high reef accretion rates. The developing “third global bleaching event”^40^ provides a clear indication of the immediacy of such threats. Actions aimed at reducing the effects of local disturbances on reefs are thus critical to provide any buffering for reefs from climate-change and to instil any capacity to maintain their accretion potential that so critically underpins the provisioning of most ecosystem services.

## Methods

Surveys were conducted during March/April 2015 on 28 reefs across the five islanded atolls of the Chagos Archipelago (numbers of survey sites in brackets): Diego Garcia (5); Peros Banhos (7), Salomon (6), Great Chagos Bank (8), and Egmont (1), as well as at one site on the submerged Blenheim Reef (Fig. 1C). A seasonally-shifting wind regime, with the predominant wind direction being from the south-east (Fig. 1B), results in marked spatial variations in wave energy around Chagos. Our site selection strategy was driven by a desire to survey sites on both the more sheltered (the south-west, western and northern margins of the atolls, and those on the more exposed margins (the north-east, east and south-eastern margins), as well as integrating sites that had been the focus of earlier ecological surveys. To enable us to classify these sites on the basis of their wave exposure regime, spatially explicit estimations of wave exposure were modelled as a function of wind speed and direction, and fetch length (i.e. the distance over open ocean that wind can travel in a specific direction unobstructed by land or reefs) (see SI Methods). Based on these model outputs (Fig. 1C) we thus classify our sites into ‘exposed’ (>1000 J m^−3^) or ‘sheltered’ (<1000 J m^−3^), this division being based on a natural break in the rank order of the data across all sites. We refer to these groupings in the text for descriptive purposes.

To quantify gross carbonate production and erosion and thus to determine net carbonate budgets (G, where G = kg CaCO_3_ m^2^ yr^−1^) we used a modified version of the Reef Budget^41^ methodology (see SI Methods). At each site, surveys were conducted at a depth of 8–10 m i.e., a little above the upper shelf break, and with replicate transects established running parallel to the reef crest, with a spacing of ~5 m between transects. With only two exceptions we collected data along 4 replicate transects at each site (the exceptions being Middle Island (n = 5) and Cannon Point (n = 3) on Diego Garcia). At the same time these surveys allowed us to collect data on substrate composition and reef rugosity as a function of the 3-dimensional surface of the reefs (see SI Methods). Based on these data we define *Acropora* spp. dominated, and *Porites/Pocillopora* spp. dominated reefs as those where these taxa contribute to >50% of coral carbonate production. To test for differences in net and gross production and erosion between sites and atolls ANOVA tests were run with, where appropriate, a Tukey post-hoc test. Principal Component Analysis (PCA) was used to explore the relationships between reef carbonate production rates and reef ecology, physical structure and exposure regime.

To assess the accretion potential of reefs, and to explore their capacity to respond to future projected regional sea-level rise rates, we converted our net production rate estimates to potential accretion rates (mm yr^−1^). We used an approach previously applied to Caribbean reefs^8^ that accounts for both framework carbonate production and sediment reincorporation from reef bioeroding taxa, but was modified to also factor for variations in accumulating framework porosity as a function of between-site variations in reef community composition^42^ (see SI Methods). Resultant reef accretion rates were compared to recent rates of sea level rise based on satellite altimetry data from the central Indian Ocean region over the period 1950–2000^43^ and 1950–2009^44^ (see SI Methods). To compare contemporary reef accretion potential to future sea-level rise trajectories we used the IPCC AR5 report projections for the period 2081–2100^35^, based on scenario RCP 4.5 but also accounting for the impacts of future wind-stress^36^.

## Additional Information

**How to cite this article**: Perry, C. T. *et al.* Remote coral reefs can sustain high growth potential and may match future sea-level trends. *Sci. Rep.* **5**, 18289; doi: 10.1038/srep18289 (2015).

## Supplementary Material

Supplementary Information
