# Supplementary material for: Remote coral reefs can sustain high growth potential and may match future sea-level trends
Source: Sci Rep. 2015 Dec 16;5:18289. doi: 10.1038/srep18289 (PMC4680928; doi:10.1038/srep18289)
Supplement: Supplementary Information [file srep18289-s1.pdf]

Supplementary methods and tables

Remote coral reefs can sustain high growth potential and may match future sea-level trends

Chris T. Perry[1*], Gary N. Murphy[1], Nicholas A.J. Graham[2,3], Shaun K. Wilson[4,5], Fraser A. Januchowski-Hartley[1], Holly K. East[1]

[1] Geography, College of Life and Environmental Sciences, University of Exeter, Exeter, U.K.

[2] Lancaster Environment Centre, Lancaster University, Lancaster LA1 4YQ, UK

[3] ARC Centre of Excellence for Coral Reef Studies, James Cook University, Townsville, QLD 4811, Australia

[4] Department of Parks and Wildlife, Kensington, Perth, Western Australia 6151, Australia.

[5] School of Plant Biology, Oceans Institute, University of Western Australia, Crawley, Western Australia 6009, Australia

*Corresponding author: c.perry@exeter.ac.uk

Supplementary methods

1. Study area

The Chagos archipelago is located in the central Indian Ocean (Lat: 5-7°S, Long: 71-73°E; and comprises five main atolls (see main Fig. 1C): Great Chagos Bank (~18,000 km^2) which is mostly submerged, but which has eight reef islands developed on its western and northern rims, Peros Banhos (~463 km^2) which has 24 reef islands around its rim, Salomon (~38 km^2) with 8 rim islands; Egmont (~40 km^2) with 2-3 islands on its rim (several islands having merged over the last decade or so), and Diego Garcia (~200 km^2) which has only 4 individual islands, but has the largest land area (2,733 ha) of any of the atolls. There are, in addition, a further ten submerged atolls and banks. Most islanded atolls have now been uninhabited for nearly 40 years, and thus the Chagos reefs are almost entirely free of direct anthropogenic impacts. Exceptions include the impacts of low (though poorly quantified) levels of illegal fishing on the outer atolls, impacts from recreational fishing around Diego Garcia (which has lower fish biomass than the other atolls [1]), and the effects of the terrestrial military development on Diego Garcia. The Chagos reefs are thus amongst the remotest in the Indo-Pacific and, in April 2010, the entire archipelago and its associated exclusive economic zone (an area of ~640,000 km^2) was officially established as a no-take marine protected area. Impacts linked to climatic change and to broad scale oceanic and meteorological disturbances thus currently represent the only serious threats to reef health and ecosystem function.

Our ecological and carbonate budget surveys were conducted during March to April 2015 on 28 reefs across each of the five islanded atolls (numbers of survey sites in brackets): Diego Garcia (5); Peros Banhos (7), Salomon (6), Great Chagos Bank (8), and Egmont (1), as well as at one site on the submerged Blenheim Reef (see main Fig. 1C). The atolls and platforms of Chagos are characterised by marked differences in wave energy regimes around their margins (see main Fig. 1C), driven by the seasonally-shifting wind regime, with the predominant wind direction being from the south-east (see main Fig. 1B). Our site selection strategy around atolls was driven by a desire to survey sites on both the more sheltered (the south-west, western and northern margins of the atolls, and those on the more exposed margins (the north-east, east and south-eastern margins), as well as integrating sites that had been the focus of earlier ecological surveys. At each site, surveys were conducted at a depth of ~10 m i.e., a little above the upper shelf break, and with replicate transects established running parallel to the reef crest, with a spacing of ~5 m between transects. With only two exceptions we collected data along 4 replicate transects at each site (the exceptions being Middle Island (n = 5) and Cannon Point (n = 3) on Diego Garcia).

2. Wave exposure modelling

To enable us to classify these sites on the basis of their wave exposure regime, spatially explicit estimations of wave exposure were modelled as a function of wind speed and direction, and fetch length (i.e. the distance over open ocean that wind can travel in a specific direction unobstructed by land or reefs). To do this we followed the same protocols previously applied in reef environments [2,3]. Fetch lengths were calculated using the USGS model[4] which uses the procedure recommended by the Shoreline Protection Manual[5]. A binary raster representing the distribution of land masses and reef crests was generated using the outputs of the Millennium Coral Reef Mapping Project at a spatial resolution of 30 m^2 [ref. 6]. Fetch lengths were generated for 16 compass directions (every 22.5°) by calculating the arithmetic mean of 5 radials spread at 3° increments around the desired wind directions. 650 km was used as a maximum limit for fetch length as this is the distance required for maximal wave conditions[7]. Hourly wind measurements from 1973 to 2001 were obtained from Diego Garcia airport (n = 219,943) and used to calculate both the probability of wind blowing from each of the 16 compass directions and also the mean velocities for each direction. Fetch lengths were then converted into wave energies based on linear wave theory and using established equations [ref2]. Based on model outputs (see main Fig. 1C) we have classified our sites into "exposed" (>1000 J m^{-3}) or "sheltered" (<1000 J m^{-3}), this division

being based on a natural break in the rank order of the data across all sites. We refer to these groupings in the text for descriptive purposes.

3. Quantifying carbonate production and erosion rates

To quantify gross carbonate production and erosion and thus to determine net carbonate production (G, where G = kg $CaCO_3$ m^2 yr^{-1}) we used a version of the ReefBudget methodology of Perry et al.[ref8] adapted and parameterised for the Indian Ocean. However, there are some importance differences: carbonate production by corals and coralline algae is calculated using geometric relationships derived from individual colony morphology, rather than calculated using rugosity at the level of the transect; and clinoid sponge bioerosion is calculated using published rates and the proportion of hard substrate under the transect line available for erosion. Sediment production by macro-bioeroders (urchins and parrotfish) is also estimated, but other aspects of sediment production and post-depositional lithification are not. Within each site, two different sets of transects were conducted, one set to census benthic carbonate producers and bioeroders, and one set to quantify parrotfish abundance and size.

Along each benthic assessment transect we measured the distance within each linear 1 m covered by each category of benthic cover beneath a 10 m guide line using a separate flexible tape. All overhangs, vertical surfaces and horizontal surfaces below the line were surveyed (i.e., if the guide line crossed over a table coral, the upper and lower surfaces of the coral, plus the benthos under the canopy, were recorded). The following groups were recorded: scleractinian corals to the genera and morphological level (e.g., *Acropora* branching, *Porites* massive etc.); crustose coralline algae (CCA) including CCA below macroalgal or soft coral cover; turf algae; fleshy macroalgae; non encrusting coralline algae (e.g., *Halimeda* sp., articulated coralline algae); sediment; bare substrate (e.g., granitic rock, limestone pavement); sediment; rubble; and other benthic organisms. Substrate rugosity was calculated as total reef surface divided by linear distance (a completely flat surface would therefore have a rugosity of 1).

3.1 Calculating carbonate production

In contrast to the original Reefbudget approach[8], we used the morphology and size of individual coral colonies in combination with genera specific skeletal density (g cm^{-3}) and linear growth rates (cm $year^{-1}$) across each transect to estimates carbonate production rates in kg $CaCO_3$ m^{-2} $year^{-1}$ (where m^2 refers to the planar surface of the reef). Where possible, we used published growth rates and skeletal densities from the Western/Central Indian Ocean, but when these were not available means of published growth rates and densities for each coral genera were used instead. These data were then combined with geometric transformations based on colony morphology to give a growth rate for each colony for the area under the transect line (taking a transect line width of 1cm): massive colonies were assumed to be hemispherical in cross-section; encrusting, foliose and plating colonies, as well as colonies of crustose coralline algae (CCA) were assumed to be growing primarily at the edge of the colony (and at 10% of this growth rate across the remainder of the colony); for branching colonies, the proportion of the colony area of growing branch tips was assumed to be growing at published rates, and the remainder of the colony at 10% of these rates. The equations used in our calculations are thus as follow:

Massive:

$$CP_i = \left(\left(g + \left(\frac{x}{\pi}\right)\right)^2 \pi - \left(\frac{x}{\pi}\right)^2 \pi\right).d$$

Encrusting etc.:

$$CP_i = 2(g.d) + 0.1g.x.d$$

Branching/corymbose etc.:

$$CP_i = (r.c_a.g.d) + (x - c_a.x).0.1g.d$$

Where CP_i = carbonate production for colony *i*, *g* = growth rate, *x* = surface length of colony, *d* = skeletal density and c_a = proportion of colony that are growing axial branches.

Measuring the linear surface of growing tips on branching corals is time-consuming. Therefore, in order to calculate the amount of each colony growing as axial branch tips, we used data previously obtained on the size of branching colonies and the length of growing tips from 405 coral colonies in northern Mozambique (293 *Acropora*, 62 *Pocillopora*, 26 *Porites*, 24 other) (ref. 9). We conducted linear regressions between colony size and length of growing tips for each genera/morphology combination for which we had greater than 20 replicates in order to calculate c_a. To calculate the production for a single transect over a year, the following equation was used:

$$CP_j = \sum_{i=1}^{n} CP_1 + CP_2 + \cdots + CP_n$$

Where CP_j is the total carbonate production of both corals and crustose coralline algae for transect *j* in kg $CaCO_3$ year^{-1}. To estimate the production rate of the reef, we then used the following equation:

$$Gprod_j = CP_j / (\frac{10000}{l})$$

Where $Gprod_j$ is the carbonate production rate of both corals and crustose coralline algae for transect *j* in kg $CaCO_3$ m^{-2} year^{-1}, and *l* is the transect length in centimetres.

3.2 Calcuating reef framework bioerosion

3.2.1. Micro- (endolithic) and Macro- (clionaid sponge, polychaetes, bivalves etc.) bioerosion

In general, macro-borer communities are less well-characterised in the Indo-Pacific than in the Caribbean. This is particularly true of clionaid sponges, which are generally cryptic and difficult to identify in the field, particularly for a non-expert, and are typically a less dominant part of the macroborer community than in the Caribbean. To this end, instead of conducting an intensive search of the substrate for clionaid sponges as described in Perry et al., (2012), we instead utilized published rates of total macrobioerosion measured at Indo-Pacific sites, alongside a census of substrate available for bioerosion from the benthic line-intercept transects. This comprises of all dead carbonate substrate available to bioeroding sponges, including that covered by macroalgae or algal turf and live coral cover and soft corals. While both live and soft corals can prevent settlement of most bioeroding sponges, live corals are often colonised by other bioeroders (particularly polychaete worms), while soft corals are often ephemeral, and removal of soft coral cover, either by predation or storms could allow settlement and establishment of bioeroders prior to regrowth. All substrate not available to bioeroders was excluded, and the following equation used:

$$\text{Microbioerosion} = S^*R^*E_m$$

$$\text{Macrobioerosion} = S^*R^*E_a$$

Where *S* is the percentage of surface area of the transect available for erosion, *R* is the rugosity of the transect, E_m is the erosion rate of microborers, and E_a is the erosion rate of macroborers in kg m^{-2} yr^{-1}.

3.2.2 Urchin bioerosion

The main agents of echinoid bioerosion on reefs belong the family Diadematidae, (*Diadema* spp., and *Echinothrix* spp.) and the genera *Echinometra*, *Echinostrephus* and *Eucidaris*. We censused urchin abundance, size and species composition within 10 x 2 m belt transects along the main benthic transect lines at each site. These census data were then combined with carbonate ingestion rates from the literature and adjusted to account for the rugosity of the substrate. A variety of techniques have been used to estimate bioerosion rates in these urchin species: including $CaCO_3$ content of the gut (e.g., ref. 10) or of their faecal pellets (e.g., ref. 11), both with or without estimations of reworked sediment, spine abrasion and gut turnover (e.g., ref. 12, 13). This makes it difficult to compare bioerosion rates derived from different studies. However, evaluating the published data on erosion rates against test size across all urchin species suggest a relatively tightly correlated plot. SI Figure 1A shows the pooled bioerosion rates relative to test size for six species of urchins across 10 studies in the Indian and Pacific Oceans, with SI Figure 1B showing the rates for *Echinometra mathaei* and the *Diadematidae*. This allows us to calculate the erosion rate (kg urchin^{-1} year^{-1}) for each individual urchin using one of the following equations:

Diadematidae bioerosion (B_D) = $(0.000001*x^{3.4192})*0.365$

Echinometra mathaei bioerosion (B_E) = $(0.0004*x^{1.9786})*0.365$

General equation for all other bioeroding species (B_G) = $(0.0001*x^{2.323})*0.365$

To calculate bioerosion by urchins in kg m^{-2} year^{-1}, we summed the erosion rates of all individual urchins within each transect (U_E), and divided by the surface areas within each transect which was calculated as planar surface area (usually 20 m^2) multiplied by rugosity.

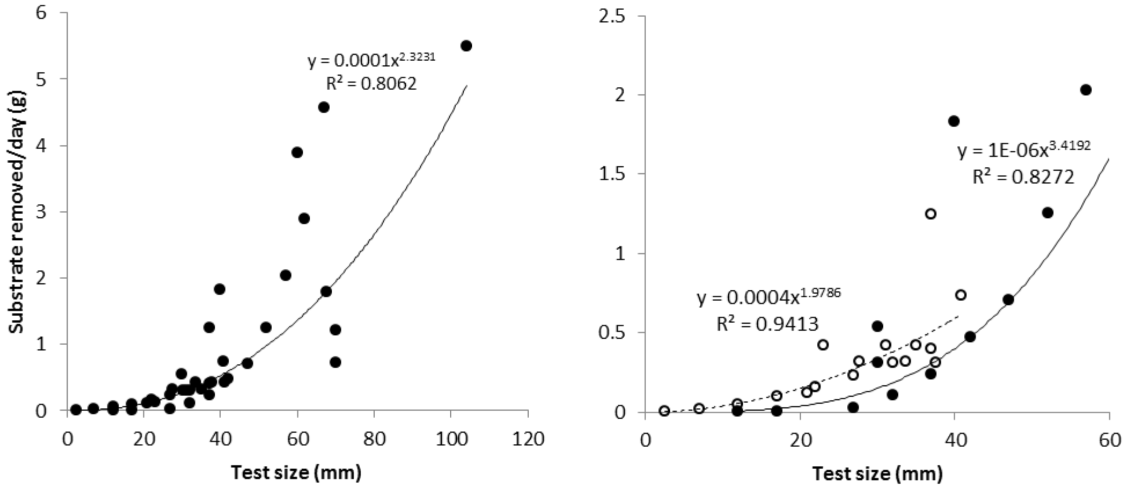

SI Figure 1 *(A) Bioerosion rates (substrate removed/day (g)) for urchins across a range of test size (Indo-Pacific data only). Data aggregated from: ref. 10, 14 – 21. (B) Bioerosion rates for Diadematidae (closed circles) and Echinometra metra (open circles).*

3.2.3. Parrotfish bioerosion

To determine the species-size-life phase abundances of bioeroding parrotfish at each site we used underwater census data for Chagos collected in 2010 and 2012[ref. 22], augmented by additional data collected in 2015. At each site four 50 m x 5 m belt transects were used (covering 1000 m^2 of reef). Time to complete transects was not held constant but adjusted according to the numbers of fish in the sampled areas. All fish surveys were completed by the same experienced observers (N.A.J.G., S.K.W or G.N.M). Biomass of individual fish was then calculated using estimated length data and length-weight relationships [23,24]) and multiplying by abundance of the species or family of the fish. To calculate parrotfish bioerosion rates by each individual fish we then used a

model based on total length and life phase to predict the bite rates (bites hr^{-1}) for each species. For species for which data was not previously available, we have made use of new observations)[9] or have used data from similar sized species with the same feeding functional group. Daily bite numbers were calculated using diurnal feeding activity reported in Bellwood[25]. Bite volume data has been obtained from published studies [25,26]. Where no bite volume data exists, we have either measured the size of individual bites *in situ* using Vernier callipers to obtain width and length of bite, or obtained from published studies estimates of bite area (e.g., Ref 27). We have then used a bite depth of 0.1 mm to obtain a conservative estimate of bite volume for *Scarus* sp. Not all bites on the substratum remove material and we have assumed, following Bellwood & Choat[28], that only bites that leave visible scars are eroding the substrate. In order to estimate the proportion of bites resulting in scars on the substratum for each size class, we extrapolated from published literature [26,29] and used the same method as above where data was missing. We used the following equation to calculate species specific erosion rates for the median value within each size class:

$$\text{Bioerosion rate (kg.ind}^{-1}\text{yr}^{-1}) = v.s_{prop}.br.d{*}365$$

Where v is bite volume (cm^3), s_{prop} is the proportion of bites leaving scars, b_r is bite rate (bites day^{-1}) and d is substratum density (kg cm^{-3}), here taken to be $1.49{*}10^{-3}$

4. Statistical treatment of data

Variation in bioerosion, and in net and gross production of carbonate, were analysed with respect to reef, atoll and exposure using analysis of variance, where reef was nested within atoll. Data were plotted and tested for normality and homogeneity of variance prior to analyses and transformed to meet these assumptions when appropriate. Significant results were further investigated with a post-hoc Tukey test. The interaction between atoll and exposure was investigated by pooling data at the reef level. Differences in the influence of different variables on net carbonate budgets between reefs were examined using correlation-based principle components analysis (PCA). Data were log(x + 1) transformed and normalized to account for skewness and to ensure all metrics were on a common scale. We overlaid eigenvectors to identify the direction and contribution of different variables to the patterns. The PCA was conducted using the prcomp function in R 3.1.1.

5. Reef accretion rates and comparisons to recent and future sea level trends

To assess the accretion potential of reefs, and to explore their capacity to respond to future projected regional sea-level rise rates, we converted our net production rate estimates to potential accretion rates (mm yr^{-1}). To do this we used an approach previously applied to Caribbean reefs [30], but modified to also factor for variations in accumulating framework porosity as a function of between-site variations in reef community composition. Specifically, we estimated the maximum accretion potential of each reef as a function of the net carbonate production rate of the site (calculated as gross production less gross erosion rate) and assumed that a proportion of the bioeroded framework (that is converted to sediment) is also reincorporated back into the accumulating reef structure. This proportion was calculated as the sum of 50% of the parrotfish-derived sediment (as a highly mobile bioeroder which defecates randomly over the reef), as well as all sediment produced by urchins and by macroborer erosion. To keep our estimates conservative we worked on the assumption that only 50% of this bioerosional sediment yield is actually incorporated back into the reef (based on data from Hubbard[31]), and excluded any sediment generation by other benthic sediment producers (e.g *Halimeda* – although this calcareous green algae was extremely rare within our survey sites). Finally, we made an allowance for variations in the porosity of the accumulating reef framework as follows: 30% for head and massive coral dominated assemblages, 70% for branched and tabular dominated assemblages, and 50% for mixed coral assemblages (based on data in Kinsey & Hopley[32]). A loss factor to account for natural framework removal through physical processes was also included based on the data of Morgan and Kench[33], such that we

assumed that 20% of the annual framework produced was removed from more sheltered reef settings, and 50% from exposed settings.

To explore the accretion potential of the Chagos reefs in relation to sea level we compared our potential accretion rate estimates to both measured recent past and future projected sea-level rise rates. Recent sea-level trends around Chagos, based on tide-gauge records, are difficult to precisely constrain due to an apparent tide-gauge offset in the Diego Garcia record around 2002 (ref. 34). However, research that has integrated these records with satellite altimetry data suggests rise rates of ~2.2 mm yr^{-1} around Diego Garcia and 1.7 mm yr^{-1} around Peros Banhos and Salomon between 1993 to 2011 (ref. 34). These rates are above those reported (~1 mm yr^{-1}) from the central Indian Ocean region over the period 1950-2000 (ref. 35), but similar to those reported for Diego Garcia (~1.75 mm yr^{-1}) over the period 1950-2009 (ref. 36). However, a defining feature of Indian Ocean sea-level trends over time, and certainly over recent decades, is one of significant intra- and inter-annual variability, in part driven by the influence of the Indian Ocean Dipole (IOD). This causes marked variations in sea-surface temperatures and wind regimes across the region, and drives considerable cross-basin changes in sea-level. Caution is thus needed in interpreting long-term trends from the relatively short term datasets presently available. Whilst Dunne[34] argue that because of such inter-annual variability the long-term regional trend is not significantly different from zero (2.22 ± 1.80 SE mm yr^{-1} (ref. 34), it is perhaps more appropriate to conservatively assume that the local rate of rise over the last half century or so has probably not exceeded the global average of ~1.7 mm yr^{-1} (ref. 37) and ~2.2 mm yr^{-1} as measured over the last ~20 years in Chagos[34]. To compare contemporary reef accretion potential to future sea level rise trajectories we used the IPCC AR5 report projections for the period 2081-2100 (ref. 37), but also accounting for the impacts of future wind-stress[38] .

References:

1. Graham, N. A. J., Pratchett, M. S., McClanahan, T. R. & Wilson, S. K. in *Coral Reefs of the United Kingdom Overseas Territories SE - 19* (ed. Sheppard, C. R. C.) **4,** 253–270 (Springer Netherlands, 2013).
2. Chollett, I. & Mumby, P. J. Predicting the distribution of *Montastraea* reefs using wave exposure. *Coral Reefs* **31,** 493–503 (2012).
3. Graham, N. A. J., Jennings, S., MacNeil, M. A., Mouillot, D. & Wilson, S. K. Predicting climate-driven regime shifts versus rebound potential in coral reefs. *Nature* **518**, 94-97 (2015).
4. Rohweder, J. Rogala, J.T., Johnson, B.L., Anderson, D., Clark, S., Chamberlin, F., Potter, D., & Runyon, K. Application of wind fetch and wave models for habitat rehabilitation and enhancement projects – 2012 update (2012) http://www.umesc.usgs.gov/management/dss/wind_fetch_wave_models_2012update.html
5. U.S. Army Corps of Engineers. Shore Protection Manual, Coastal Engineering Research Center, Fort Belvoir, Virginia (1984).
6. Andréfouët, S. *et al.* Global assessment of modern coral reef extent and diversity for regional science and management applications: a view from space. in *Proceedings of the 10th International Coral Reef Symposium, Okinawa, Japan, 28 June-32nd July 2004* (eds. Suzuki, Y. et al.) (Japanese Coral Reef Society, 2006).
7. Hill, N. A. *et al.* Quantifying wave exposure in shallow temperate reef systems: Applicability of fetch models for predicting algal biodiversity. *Mar. Ecol. Prog. Ser.* **417,** 83–95 (2010)
8. Perry, C. T. *et al.* Estimating rates of biologically driven coral reef framework production and erosion: A new census-based carbonate budget methodology and applications to the reefs of Bonaire. *Coral Reefs* **31,** 853–868 (2012).
9. Januchowski-Hartley, F.A., McClanahan, T.R., Cossa, D., Perry, C.T. (in prep.) Isolation and community composition drive reef growth in East Arica.
10. Conand, C., Chabanet, P., Cuet, P., & Letourneur, Y. The carbonate budget of a fringing reef in La Reunion Island (Indian Ocean): sea urchin and fish bioerosion and net calcification. In *Proceedings of the 8th International Coral Reef Symposium* (eds. Lessios, H. A., & Macintyre, I. G., Vol. 1, Smithsonian Tropical Research Institute, Panama pp. 953-958 1987).
11. Glynn, P., Wellington, G. & Birkeland, C. Coral reef growth in the Galapagos: limitation by sea urchins. *Science* **203,** 47–49 (1979).
12. Scoffin, T. P. *et al.* Calcium-carbonate budget of a fringing-reef on the west-coast of Barbados. 2. Erosion, sediments and internal structure. *Bull. Mar. Sci.*, **30**, 475-508. (1980)

13. Griffin, S. P., García, R. P. & Weil, E. Bioerosion in coral reef communities in southwest Puerto Rico by the sea urchin *Echinometra viridis*. *Mar. Biol.* **143,** 79–84 (2003).
14. Russo, A.R. Bioerosion by two rock boring echinoids (*Echinometra mathaei* and *Echinostrephus aciculatus*) on Enewetak Atoll, Marshall Islands. *J. Mar. Res.* **38**, 99-110 (1980).
15. Downing, N. & El-Zahr, C. Gut evacuation and filling rates in the rock-boring sea urchin, *Echinometra mathaei*. *Bull. Mar. Sci.* **41,** 579–584 (1987).
16. McClanahan, T. R. & Mwashote, B. M. Changes in Kenian coral reef community structure and function due to exploitation. *Hydrobiologia* **166,** 269–276 (1988).
17. Bak, R. Patterns of echinoid bioerosion in two Pacific coral reef lagoons. *Mar. Ecol. Prog. Ser.* **66,** 267–272 (1990).
18. Mokady, O., Lazar, B. & Loya, Y. Echinoid bioerosion as a major structuring force of red sea coral reefs. *Biol. Bull.* **190,** 367–372 (1996).
19. Mills, S. C., Peyrot-Clausade, M. & France Fontaine, M. Ingestion and transformation of algal turf by *Echinometra mathaei* on Tiahura fringing reef (French Polynesia). *J. Exp. Mar. Bio. Ecol.* **254,** 71–84 (2000).
20. Carreiro-Silva, M. & McClanahan, T. R. Echinoid bioerosion and herbivory on Kenyan coral reefs: The role of protection from fishing. *J. Exp. Mar. Bio. Ecol.* **262,** 133–153 (2001).
21. Herrera-Escalante, T., López-Pérez, R. a. & Leyte-Morales, G. E. Bioerosion caused by the sea urchin *Diadema mexicanum* (Echinodermata: Echinoidea) at Bahías de Huatulco, Western Mexico. *Rev. Biol. Trop.* **53 Suppl 3,** 263–273 (2005).
22. Graham, N. A. J. & Mcclanahan, T. R. The Last Call for Marine Wilderness? *Bioscience* **63,** 397–402 (2013).
23. Letourneur, Y. Length-weight relationship of some marine fish species in Reunion Island, Indian Ocean. *Naga*, **21**, 37-39 (1998).
24. Froese, R. & Pauly, D. FishBase (www.fishbase.org). World wide web electronic publication (2012)
25. Bellwood, D. Direct estimate of bioerosion by two parrotfish species, *Chlorurus gibbus* and *C. sordidus*, on the Great Barrier Reef, Australia. *Mar. Biol.* 419–429 (1995).
26. Ong, L. & Holland, K. N. Bioerosion of coral reefs by two Hawaiian parrotfishes: species, size differences and fishery implications. *Mar. Biol.* **157,** 1313–1323 (2010).
27. Lokrantz, J., Nyström, M., Thyresson, M. & Johansson, C. The non-linear relationship between body size and function in parrotfishes. *Coral Reefs* **27,** 967–974 (2008).
28. Bellwood, D. R. & Choat, J. H. A functional analysis of grazing in parrotfishes (family Scaridae): the ecological implications. *Environ. Biol. Fishes* **28,** 189–214 (1990).
29. Bruggemann, J., van Kessel, A., van Rooij, J. & Breeman, A. Bioerosion and sediment ingestion by the Caribbean parrotfish *Scarus vetula* and *Sparisoma viride*: implications of fish size, feeding mode and habitat use. *Mar. Ecol. Prog. Ser.* **134,** 59–71 (1996).
30. Perry, C. T. *et al.* Caribbean-wide decline in carbonate production threatens coral reef growth. *Nat. Commun.* **4,** 1402 (2013).
31. Hubbard, D. K. Depth and species-related patterns of Holocene reef accretion in the Caribbean and western Atlantic: a critical assessment of existing models. *Spec. Publ. Int. Assoc. Sedimentol.* **40**, 1–18 (2008).
32. Kinsey, D.W. & Hopley, D. The significance of coral reefs as global carbon sink-response to greenhouse. *Palaeogeogr. Palaeoclimatol. Palaeoecol.* **89**, 363–377 (1991).
33. Morgan KM & Kench PS A detrital sediment budget of a Maldivian reef platform. *Geomorphology* **222**, 122–131 (2014).
34. Dunne, R. P., Barbosa, S. M. & Woodworth, P. L. Contemporary sea level in the Chagos Archipelago, central Indian Ocean. *Global and Planetary Change*. 82-83: 25-37.
35. Church, J.A., White, N.J. & Hunter, J.R. Sea-level rise at tropical Pacific and Indian Ocean islands. *Global and Planetary Change* **53**, 155–168 (2006).
36. Palanisamya, H. *et al.* Regional sea level variability, total relative sea level rise and its impacts on islands and coastal zones of Indian Ocean over the last sixty years. *Global and Planetary Change* **116**, 54–67 (2014).
37. Church, J.A. *et al.* Sea Level Change. In: *Climate Change 2013: The Physical Science Basis. Contribution of Working Group I to the Fifth Assessment Report of the Intergovernmental Panel on Climate Change* [Stocker, T.F., D. Qin, G.-K. Plattner, M. Tignor, S.K. Allen, J. Boschung, A. Nauels, Y. Xia, V. Bex and P.M. Midgley (eds.)]. Cambridge University Press, Cambridge, United Kingdom and New York, NY, USA (2013).
37. Timmermann, A., S. McGregor, & Jin, F. F. Wind effects on past and future regional sea level trends in the southern Indo-Pacific, *J. Clim.* **23**, 4429–4437 (2010)

Supplementary Table S1

Site	% *Acropora*	% *Porites*	% *Pocillop.*	% other corals	Cover cover %	Gross G	Erosion G	Net G	Accretion rate (mm yr^{-1})	Exposure (j m^{-3})
**Diego Garcia**										
Cannon Point<br>S 07°15’27.3”; E 072°22’14.8”	18	11	23	48	16.5	2.72	2.12	0.60	1.32	673
Middle Island<br>S 07°13’35.3”; E 072°24’28.02	37	31	9	23	36.4	8.04	4.00	4.04	1.51	678
Barton Point<br>S 07°14’00.3”; E 072°26’23.9”	36	33	3	28	42.9	9.77	2.97	6.79	1.64	1065
East Island<br>S 07°13’25.0”; E 072°25’16.3”	15	27	22	36	10.6	2.02	1.45	0.57	1.27	1050
Horsborough Bay<br>S 07°13’33.9”; E 072°24’29.1”	6	20	6	68	27.7	6.06	1.67	4.74	1.7	1005
**Peros Banhos**										
Ile Poule<br>S 05°23’52.2”; E 071°44’57.6”	59	18	3	20	40.9	11.71	3.75	8.23	2.29	797
Ile Gabrielle<br>S 05°25’15.0”; E 071°44’50.0”	56	15	3	26	30.5	7.09	2.83	4.31	2.18	884
Ile Diamante<br>S 05°14’46.8”; E 071°46’10.1”	29	24	5	42	35.3	7.46	2.59	4.87	2.04	652
Ile de la Passe<br>S 05°14’14.8”; E 071°48’57.4”	48	23	4	25	37.1	10.34	3.36	6.98	2.48	702
Ile Fouquet<br>S 05°27’40.3”; E 071°46’23.5”	16	48	16	20	24.7	7.55	3.36	4.19	2.05	1265
Petite Coquillage<br>S 05°20’22.9”; E 071°58’40.0”	15	54	9	22	39.7	10.15	7.44	2.7	1.95	1405
Grand Coquillage<br>S 05°22’20.9”; E 071°58’31.2”	17	60	6	17	34.9	11.42	1.64	9.76	2.28	1389
**Salomon**										
Ile Anglaise – south<br>S 05°20’22.7”; E 072°12’49.1”	75	12	2	11	24.3	7.81	3.93	3.87	2.57	510
Ile Anglaise – middle<br>S 05°19’45.0”; E 072°13’10.5”	48	23	7	22	22.1	7.88	3.96	3.91	2.71	514
Ile Anglaise – north<br>S 05°18’58.7”; E 072°13’32.1”	31	49	7	13	28.1	9.57	1.80	7.77	2.38	623
Ile de Passe<br>S 05°14’14.8”; E 071°48’57.4”	56	22	10	12	33.1	9.16	4.04	5.12	2.32	780
Ile Takamaka<br>S 05°19’80.0”; E 072°16’50.0”	10	44	32	14	6.6	1.64	1.52	0.12	1.3	1690
Ile du Sel<br>S 05°21’37.4”; E 072°13’36.4”	12	45	9	34	13.6	3.75	3.50	0.24	1.81	1404
**Blenheim**										
Blenheim, west side<br>S 05°12’11.4”; E 072°27’16.7”	52	2	20	26	30.4	7.68	3.64	4.04	1.91	688
**Gt Chagos Bank**										
Nelson, north<br>S 05°40’46.9”; E 072°18’59.7”	0	45	10	45	3.6	1.86	6.88	-5.03	2.27	545
Eagle - northern end<br>S 06°10’26.4”; E 071°19’60.0”	24	64	4	8	21.3	4.92	2.26	2.66	1.65	795
Eagle - southern end<br>S 06°12’10.1”; E 071°18’44.6”	6	57	7	30	19.3	5.55	3.28	2.26	1.91	761
Eagle - middle site<br>S 06°11’18.0”; E 071°19’10.5”	16	54	7	23	12.9	3.27	4.08	-0.81	1.5	770
Danger Island<br>S 06°23’30.0”; E 071°14’40.0”	13	38	21	28	10.1	3.72	3.98	-0.26	1.95	591
Middle Brother<br>S 06°09’21.6”; E 071°30’40.0”	35	32	1	32	34.6	8.06	2.60	5.47	2.04	1193
South Brother - west end<br>S 06°10’25.3”; E 071°32’18.3”	65	14	3	18	40.7	10.07	5.46	4.59	2.11	1212

South Brother - east end<br>S 06º10'30.4"; E 071º32'34.0"	41	21	7	31	43.4	10.65	3.87	6.78	1.98	1288
**Egmont**										
Egmont<br>S 06º38'44.5"; E 071º22'12.0"	41	16	4	39	29.9	6.88	2.06	4.81	1.9	1133
